# Is the high-performance thermoplastic polyetheretherketone indicated as a clasp material for removable dental prostheses?

**DOI:** 10.1007/s00784-020-03603-y

**Published:** 2020-10-07

**Authors:** Danka Micovic, Felicitas Mayinger, Sebastian Bauer, Malgorzata Roos, Marlis Eichberger, Bogna Stawarczyk

**Affiliations:** 1 Department of Prosthetic Dentistry, University Hospital, LMU Munich, Goethestraße 70, 80336 Munich, Germany; 2 Department of Biostatistics, Epidemiology, Biostatistics and Prevention Institute, University of Zurich, Hirschengraben 84, 8001 Zurich, Switzerland

**Keywords:** PEEK, Cobalt-chrome-molybdenum, Clasp, Removable dental prosthesis, Retention force

## Abstract

**Objectives:**

To investigate the retention force of polyetheretherketone (PEEK) removable dental prosthesis clasps in comparison with a cobalt-chrome-molybdenum control group after storage in artificial saliva.

**Materials and Methods:**

Clasps were milled (Dentokeep (PEEKmilled1), NT digital implant technology; breCAM BioHPP Blank (PEEKmilled2), bredent), pressed (BioHPP Granulat for 2 press (PEEKpressed), bredent), or cast (remanium GM 800+ (cobalt-chrome-molybdenum), Dentaurum); *N* = 60, *n* = 15/subgroup. Retention force was examined 50 times/specimen in a pull-off test using the universal testing machine (Zwick 1445), where pull-off force was applied with a crosshead speed of 5 mm/minute until the maximum force dropped by 10%, at different aging levels: (1) initial, after storage in artificial saliva for (2) 90 and (3) 180 days. Statistical analysis was performed using one-way ANOVA followed by post hoc Scheffé-test and mixed models (*p* < 0.05).

**Results:**

Cobalt-chrome-molybdenum presented the highest retention force. No differences were observed between polyetheretherketone materials. Cobalt-chrome-molybdenum showed a significant decrease of its values after artificial aging, while polyetheretherketone materials presented similar results over the course of aging. Regarding a repetitive insertion and removal, even though PEEKmilled2 and cobalt-chrome-molybdenum showed an initial increase, ultimately, a decrease in retention force was observed for all tested groups.

**Conclusions:**

Although the control group showed significantly higher results, the retention force of polyetheretherketone materials indicate a potential clinical application. Neither the manufacturing process nor artificial aging showed an impact on the retention force of polyetheretherketone clasps.

**Clinical relevance:**

Mechanical properties of novel removable dental prosthesis clasp materials devised to meet the growing esthetic demands of patients need to be investigated to ensure a successful long-term clinical application.

## Introduction

Due to recent leaps in implant and restorative dentistry, fixed dental prostheses (FDPs) allow highly esthetic results for a wide majority of patients. In some cases, an extensive replacement of missing teeth does, however, still require treatment with removable dental prostheses (RDPs) because of reduced health, challenging anatomical situations, physiology, or financial reasons [1].

Clasps can be used as retention elements to attach a prosthesis to the remaining teeth, thus ensuring functional stability during enunciation and mastication. In the course of time, a wide variety of clasps have been designed to tailor to various indications. Clasps traditionally consist of a retentive arm that passes over the prosthetic equator and comes to a rest in an undercut, while the reciprocal arm undertakes the task of opposing lateral forces during insertion and removal [2]. The depth of the undercut as well as the elastic modulus of the clasp material directly affects the retention of RDPs [3].

Metal alloy has for a long time been the material of choice for RDP clasps, as its outstanding mechanical properties are well documented [4–12]. The alloy most commonly used is cobalt-chrome-molybdenum (CoCrMo) [13]. Numerous studies have observed significantly higher retention load values of CoCrMo clasps than seen for alternative materials such as titanium [11, 12]. With ever rising esthetic demands, research activities have focused on tackling the main drawback of alloy clasps: their metallic color. To eliminate the esthetically disadvantageous retentive arm, lingual retentions or rotational paths were investigated as alternatives to conventional clasp designs [14, 15]. Others aimed to modify the alloy claps itself by etching and veneering said materials with tooth-colored resin composite [16].

One relatively new approach is to manufacture clasps of a tooth-colored thermoplastic material, such as polyoxymethylene [17], polycarbonate and polyamide [3], or polyaryletherketone (PAEK) [18]. The term “PAEK” comprises a number of closely related high-performance thermoplastics, from polyetheretherketone (PEEK) over polyetherketoneketone (PEKK) to aryl ketone polymer (AKP), that convince with notable mechanical properties and manifold applications in the field of dentistry [19, 20]. In prosthodontics, PEEK is employed as a framework material for fixed and removable dental prostheses and the manufacturing of clasps and implant abutments [21, 22]. PEEK may also hold a promising future in dental implantology. While unmodified PEEK is less osseoconductive and bioactive than titanium [23], dental implants made from PEEK have been shown to exhibit less stress shielding when compared with titanium [21]. Studies have furthermore observed a high biocompatibility and chemical stability of PEEK to both organic and inorganic chemicals [24, 25]. This finding is of special importance for patients prone to allergies. In a dental technical laboratory, PEEK can be processed by pressing the extruded material with a special vacuum-pressing device. For this purpose, PEEK is used either as pellets or in its granular form. Computer-aided design (CAD) and computer-aided manufacturing (CAM) technology enable an alternative manufacturing process by milling PEEK restorations from prepressed blanks. The industrial pre-pressing of blanks has been observed to increase the stability and reliability of PEEK restorations [26]. While all these fabrication methods allow using the same raw PEEK material, results of mechanical stress tests for these materials are very limited [19, 26] and the available literature varies considerably in terms of the investigated prosthetic applications.

Therefore, the aim of this study was to examine the retention force of RDPs’ clasps made from three different PEEK materials in comparison with a CoCrMo control group after storage in artificial saliva. One important aspect when conducting an in vitro study is the close approximation to the clinical situation. To test the specimens’ long-term performance, artificial aging was thus included in the study design [27]. The study tested the null hypothesis that neither the clasp material, the different manufacturing processes for the PEEK clasps, artificial aging, nor a repetitive insertion and removal of the clasps on an abrasion-resistant CoCrMo model showed an impact on the retention force.

## Materials and methods

**Fig. 1:**
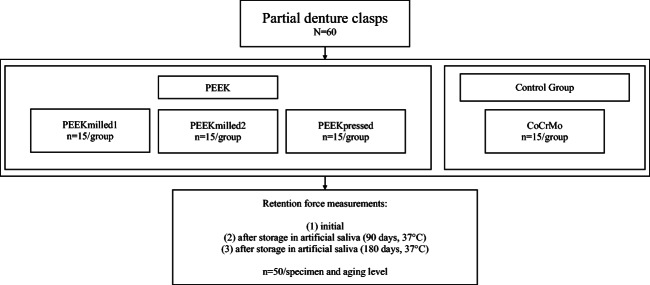
Study design

The retention force of RDP clasps made from three differently manufactured PEEK materials (Dentokeep (abbreviation: PEEKmilled1), NT digital implant technology, Karlsruhe, Germany; breCAM BioHPP Blank (PEEKmilled2) and BioHPP Granulat for 2 press (PEEKpressed), bredent, Senden, Germany) and a CoCrMo alloy (control group; remanium GM 800+ (CoCrMo), Dentaurum, Ispringen, Germany) was examined in a pull-off test at different aging levels (Table 1, Fig. 1).

**Table 1:** Materials, abbreviations, Young’s modulus, manufacturers, compositions, and lot. no. used

Material	Abbreviations	Young’s modulus	Manufacturers	Compositions	Lot. no.
Dentokeep	PEEKmilled1	4 GPa	NT digital implant technology, Karlsruhe, Germany	Polyether ether ketone, inorganic fillers (20%)	11DK18001
breCAM BioHPP Blank	PEEKmilled2	4 GPa	bredent, Senden, Germany		380149
BioHPP Granulat for 2 press	PEEKpressed	4 GPa			379806
remanium GM 800+	CoCrMo	230 GPa	Dentaurum, Ispringen, Germany	Co (58.3%), Cr (32.0%), Mo (6.5%), W (1.5%), Si (1.0%)	816
Zeno PMMA cast Disc		2.4 GPa	Wieland Dental Pforzheim, Germany	Polymethylmethacrylate	1304

### Specimen fabrication

**Fig. 2:**
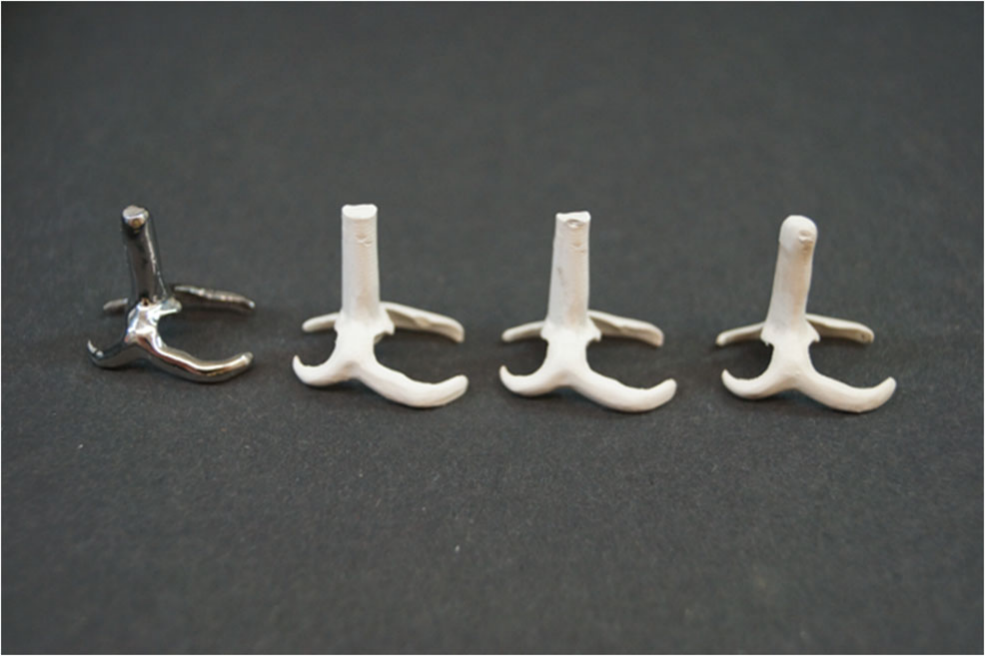
RDP clasp specimens made of CoCrMo, PEEKmilled1, PEEKmilled2, and PEEKpressed

To produce 15 RDP clasp specimens from each material (*N* = 60; *n* = 15/subgroup; Fig. 2), a hollow form for a Bonwill clasp was prepared between the second pre- and first molar of a dental arch model (Frasaco Mandible 119, A-3, Franz Sachs & Co, Tettnang, Germany). By casting (Globucast, Krupp AG, Essen, Germany) with the lost-wax technique (Finowax, DT, Bad Kissingen, Germany), a master clasp was fabricated from CoCrMo (remanium GM 800 +). To allow for a later positioning in the pull-off test, the casting channel, which had been positioned in the insertion direction of the Bonwill clasp, was cut at a height of 15 mm. The model specimen was subsequently air-particle abraded (basis Quattro IS, Renfert, Hilzingen, Germany) with 110 μm Al_2_O_3_ (Korox 110, Bego, Bremen, Germany) at 0.2 MPa and polished with a silicone polisher and a polishing brush (Komet, Gebr. Brasseler GmbH & Co. KG, Lemgo, Germany). A master STL file (Table 2) was then created by scanning (Ceramill map 300, Amann Girrbach, Koblach, Austria) the model CoCrMo specimen.

**Table 2:** Dimensions of the Bonwill clasp

	Length (mm)	Height (mm)	Width (mm)	Undercut (mm)
Retentive arm, overall (external dimension)	19.0			
Reciprocal arm, overall (external dimension)	16.2			
Retentive arm, short (inner dimension)	4.9	2.33	1.76	0.75
Retentive arm, long (inner dimension)	10.5	2.9	1.72	1.0
Reciprocal arm, short (inner dimension)	5.5	1.79	1.73	
Reciprocal arm, long (inner dimension)	8.7	2.91	1.89	
Support		2.0	4.8	
Connector			4.5 × 4.92	

Retentive arm (buccal), reciprocal arm (lingual), short arm (premolar), long arm (molar)

Employing CAM software (Zenotec CAM, V2.2.017, Wieland Dental + Technik, Pforzheim, Germany) and a milling machine (i-Mes 4030, Wieland Dental + Technik), PMMA (Zeno PMMA cast Disc, Wieland Dental + Technik; *n* = 30) and PEEK (Dentokeep and breCAM; *n* = 15/subgroup) clasps were manufactured.

**Fig. 3:**
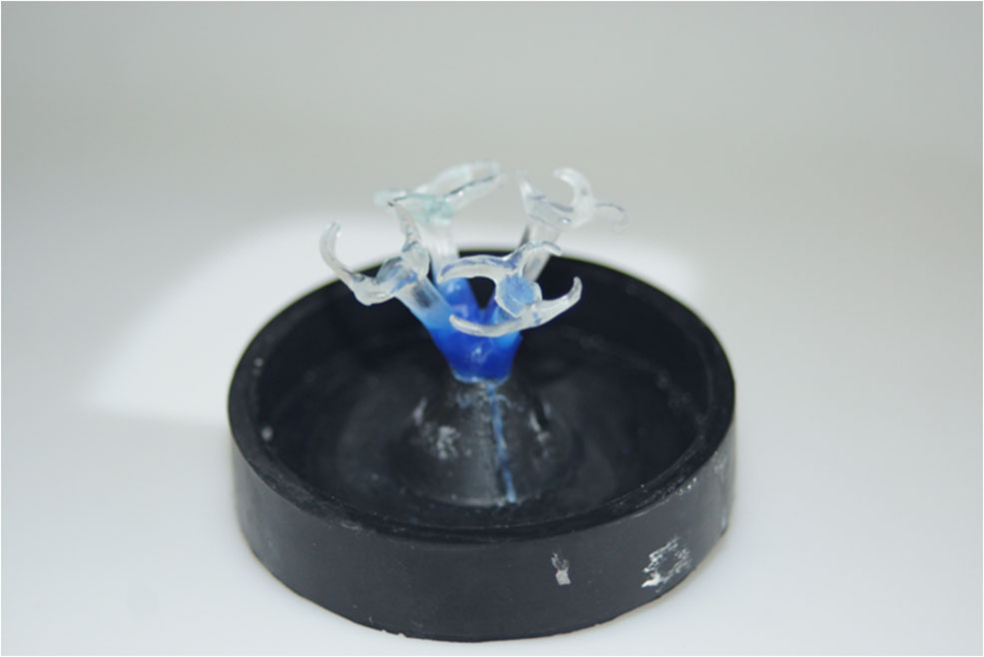
PMMA clasps prior to embedding during the manufacturing process of PEEKpressed and CoCrMo specimens

Afterwards, PMMA clasps were embedded (Brevest for 2 press, bredent) in a muffle according to the manufacturer’s instruction (Fig. 3). The investment ring was heated at 8 °C/s to 630 °C (ARCA 20, Schütz Dental, Rosbach, Germany) and then cooled to 400 °C. Subsequently, the pre-heated muffle was filled with Granulat and kept in the preheating oven for 20 min. As the next step, Granulat was pressed at 0.45 MPa under vacuum (for 2 press, bredent).

The remaining PMMA clasps were embedded (JET2000, Siladent, Dr. Böhme & Schöps GmbH, Goslar, Germany) in a similar workflow, before the investment ring was heated at 6 °C/s to 900 °C (KaVo EWL 5636, KaVo Dental GmbH, Biberach/Riß, Germany). CoCrMo specimens (remanium GM 800 +) were then cast at 1410 °C with a pressure of 0.45 MPa (Globucast).

After cooling, the investment material was removed from PEEKpressed and CoCrMo specimens using a blasting unit (Fine-blaster type FG 3, Sandmaster, Zofingen, Switzerland) with 105 μm Al_2_O_3_ (Hasenfratz) at a pressure of 0.2 MPa.

Clasps were subsequently polished with a silicone polisher and a polishing brush (Komet). High gloss was achieved with a goat hairbrush and buffing wheel using polishing paste (Universal-Polierpaste, Ivoclar Vivadent, Ellwangen, Germany). The fit of the clasp specimens on CoCrMo models was adjusted and verified with occlusion foil (Hanel Okklusions-Folie 12 μm, Coltène/Whaledent AG, Altstätten, Switzerland).

### Retention force measurement

Retention force was determined at three different aging levels:

1. Initial
2. After storage in artificial saliva for 90 days at 37 °C in an incubator (Hera Cell 150, Heraeus, Hanau, Germany)
3. After storage in artificial saliva for 180 days at 37 °C in an incubator (Hera Cell 150)

Artificial saliva was prepared according to Fusayama Meyer et al. [28] (components: potassium chloride [0.4 g/l], sodium chloride [0.400 g/l], calcium chloride dihydrate [0.906 g/l], monosodium phosphate dihydrate [0.690 g/l], sodium sulfide nonahydrate [0.005 g/l], urea [1.000 g/l]; pH = 4.7) and replaced every 14 days.

**Fig. 4:**
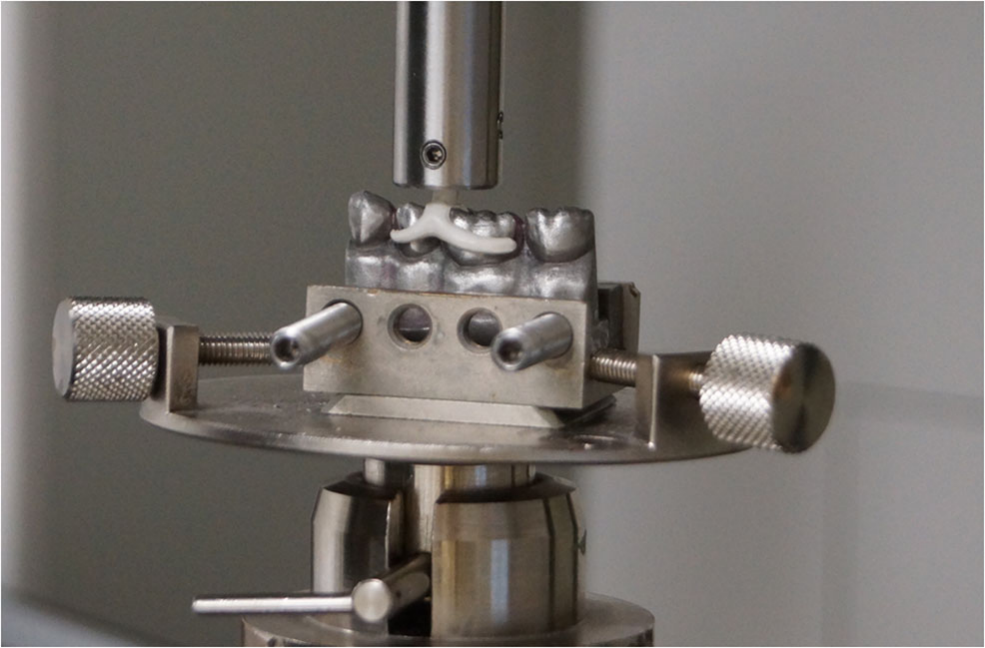
Retention force measurement (Zwick 1445, Zwick GmbH & Co. KG)

Casting channels/connectors were inserted in an individually manufactured stainless steel adapter (SD Mechatronik GmbH, Feldkirchen, Germany; Fig. 4) after CoCrMo models were positioned in the insertion/removal direction of the Bonwill clasp. Using the universal testing machine (Zwick 1445, Zwick GmbH & Co. KG, Ulm, Germany), pull-off force was applied in direct extension of the casting channel/connector with a crosshead speed of 5 mm/min until the maximum force dropped by 10%. At the three different aging levels, 50 retention force measurements were performed for each clasp at each aging level.

### Statistical analysis

A power analysis using the retention force values of the control group (163 ± 55 N) had been computed using nQuery Advisior (Version 6.04.10, Statistical Solutions, Saugaus Mass, USA) prior to performing this study. Employing a two-group *t* test with a significance level of α = 0.05 showed that a sample size of 15 in each group would have a power of 97% to detect a difference of 81.5 N. Under identical conditions, a Bonferroni correction would have a power of 92%.

A statistical evaluation of the data was performed using descriptive analysis followed by Kolmogorov-Smirnov for testing the violation of normal distribution. To determine the influence of the material and the aging level on the retention force, one-way ANOVA followed by Scheffé post hoc test was computed. Because each clasp was measured 50 times, leading to dependent measurements, linear mixed models were applied to determine global retention force values within the tested groups and potential changes of these values at different aging levels.

All *p* values below 0.05 were construed as statistically significant. Data were analyzed with SPSS version 25.0 (IBM, Armonk, NY, USA).

## Results

The results of the descriptive analyses are presented in Table 3. Parametric tests were performed, as no violation of normality assumption was indicated.

**Table 3:** Descriptive statistics for the retention force [N] of the different clasp materials at varying aging levels

Aging level	PEEKmilled1		PEEKmilled2		PEEKpressed		CoCrMo	
	Mean ± SD	95% CI	Mean ± SD	95% CI	Mean ± SD	95% CI	Mean ± SD	95% CI
(1) Initial	58.1 ± 18.8^a^	[47.6; 68.5]	43.9 ± 22.6^a^	[31.1; 56.4]	50.8 ± 17.9^a^	[40.8; 60.8]	163 ± 55.2^b^	[132; 193]
(2) After storage in artificial saliva (90 days, 37 °C)	43.0 ± 14.4^a^	[35.0; 51.0]	40.3 ± 20.4^a^	[29.0; 51.6]	45.6 ± 14.9^a^	[37.3; 53.9]	127 ± 40.4^b^	[104; 149]
(3) After storage in artificial saliva (180 days, 37 °C)	36.4 ± 9.50^a^	[31.1; 41.7]	33.5 ± 13.3^a^	[26.0; 40.9]	35.7 ± 13.2^a^	[28.3; 43.0]	102 ± 29.3^b^	[86.2; 119]

^abc^Different letters present significant differences between the different materials within one aging level

The choice of clasp material presented a significant impact on the retention force, with the control group showing higher values than the three PEEK materials (*p* < 0.001). No differences in retention force were observed between different PEEK materials (*p* = 0.412–0.607).

**Fig. 5:**
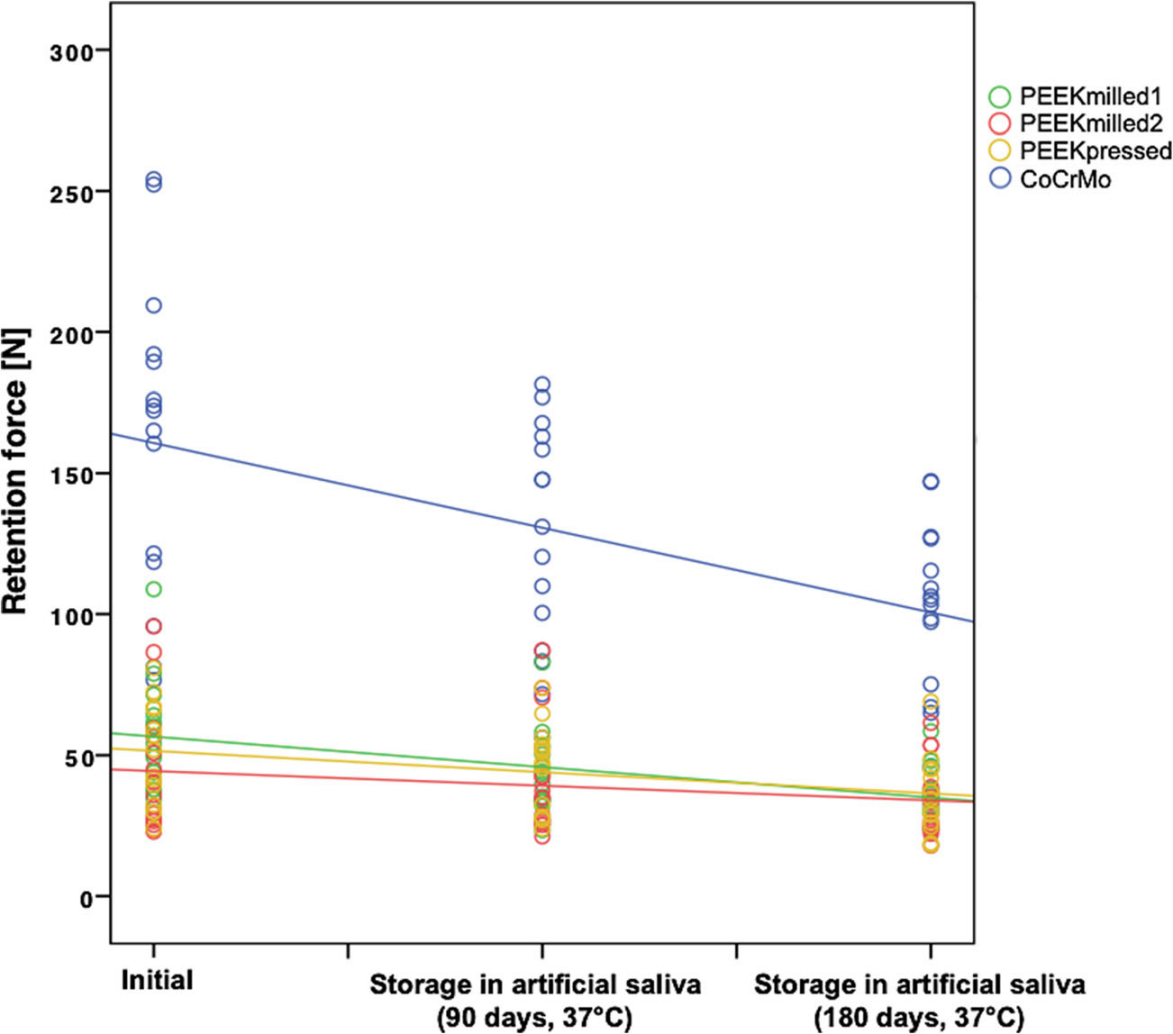
Graphical illustration of the retention force [N] of the different clasp materials at the three aging levels

Artificial aging showed an influence on the retention force of the different materials, with the control group presenting a significant decrease of its values (*p* < 0.01, Fig. 5). There is no evidence that PEEK materials show any decrease over the course of aging (*p* = 0.236–0.401).

The repetitive insertion and removal of the clasps led to a reduction of the retention force of PEEKmilled1 (*p* < 0.001–0.048; Table 4) and PEEKpressed (*p* < 0.001) specimens at all aging levels. For PEEKmilled2 and CoCrMo, an increase of retention force was observed initially (*p* < 0.001), before values decreased with a repetitive insertion and removal of the RDP clasps at the subsequent aging levels (*p* < 0.001–0.199).

**Table 4:** Influence of a repetitive insertion and removal on the retention force [N] of the different clasp materials at varying aging levels

Aging level	PEEKmilled1		PEEKmilled2		PEEKpressed		CoCrMo	
	Mean ± SD	95% CI	Mean ± SD	95% CI	Mean ± SD	95% CI	Mean ± SD	95% CI
(1) Initial	− 1.7^c^	[− 3.4; − 0.02]	7.5^b^	[6.4; 8.6]	− 4.8^c^	[− 6.0; − 3.5]	14.8^c^	[11.3; 18.3]
(2) After storage in artificial saliva (90 days, 37 °C)	− 9.1^b^	[− 9.9; − 8.2]	− 0.5^b^	[− 1.2; − 0.2]	− 1.3^b^	[− 1.8; − 0.7]	− 11.8^b^	[− 14.4; − 9.1]
(3) After storage in artificial saliva (180 days, 37 °C)	− 0.7^a^	[− 1.2; − 0.2]	− 6.4^a^	[− 7.3; − 5.5]	− 4.3^a^	[− 5.0; − 3.6]	− 6.8^a^	[− 8.7; − 4.8]

^abc^Different letters present significant differences between aging levels within one material

## Discussion

The aim of this study was to examine the retention force of RDP clasps made from different PEEK materials in comparison with a CoCrMo control group after storage in artificial saliva to imitate clinical conditions. The null hypothesis had to be rejected, as the results showed all tested parameters to affect the retention force.

When regarding the choice of clasp material, the control group showed superior retention values compared to the three PEEK materials. These results are in line with previous examinations investigating the retentive force and fatigue resistance of both PEEK and CoCr clasps [18, 29, 30]. Even though PEEK clasps presented lower values, they might provide enough retention for a clinical usage, as they exceed the suggested retention force of 5–10 N per clasp [31, 32]. As excessive retentive forces can overstrain the remaining abutment teeth, especially in periodontally compromised dentitions [33], PEEK materials could represent a valid alternative. As all PEEK materials showed similar results over the course of aging, the manufacturing process does not seem to hold an influence on the resulting mechanical properties. In the present study, two PEEK materials were milled using CAD/CAM technology, while one material was pressed. As most dental laboratories nowadays have access to high-end milling machines, this elegant process regarded to be less time-consuming and prone to manual mistakes should be preferred [34].

Artificial aging also presented an impact on the retention force. The control group showed a high decrease of its values, while PEEK clasps presented similar results before and after the aging process. A high decrease in the retention force of the control group can be explained by alloy corrosion taking place in wet environments, which has previously been reported to lead to a reduced fatigue strength of CoCr [35]. While the three PEEK materials also presented a decline in retention force, this was not significant. These results are consistent with a previous study investigating the behavior of PEEK during artificial aging with different saliva solutions that reported the thermoplastic to show a great structural stability and little or no impact of varying pH values on its nanomechanical properties [36].

The repetitive insertion and removal of the clasps led to a reduction of the retention force of PEEKmilled1 and PEEKpressed specimens at all aging levels. For PEEKmilled2 and CoCrMo, an increase of retention force was observed initially, before values decreased with a repetitive insertion and removal of the RDP clasps at the subsequent aging levels. An initial increase in retention force might be explained by abrasion phenomena of both the model and clasps resulting in an improved fit of the clasps and in consequence, an increased retention force. A previous examination investigating the retentive force of thermoplastic resins and cobalt-chrome over a simulation period of 10 years reported similar findings with an initial increase in values during the first period of cycling that was later on substituted by a continuous decrease [18]. The elastic modulus plays an important role in fatigue testing, as a material with a high elastic modulus is able to assume its prior structure without permanent deformation. CoCrMo, which possesses a high elastic modulus of 220 GPa [37], should thus in theory be less prone to a decrease in retention force due to a repetitive insertion and removal of the clasps than PEEK, which only holds an elastic modulus of around 4 GPa [38]. In contrast to this idea, a recent study observed polymer-based clasps to act more consistently over a prolonged aging process, which included cycles of repeated insertion and removal along both ideal and non-ideal paths in artificial saliva, while exhibiting inferior retention forces in comparison to conventional CoCr clasps [20].

Regarding clinical implications, PEEK materials might therefore represent the material of choice for anterior abutment teeth that possess little anatomical undercut and in consequence require little deformation during insertion and removal, while CoCrMo could be the material of choice for the posterior regions, where molars provide a large retentive area and high masticatory forces demand superior retentive capacities and functional stability [2]. Individual patient situations might thus call for individualized treatment planning regarding the choice of clasp material.

As of today, only few reports about PEEK’s behavior in clinical conditions are available. According to one recently published case report with a 2-year follow-up period, PEEK shows promising results, as few color and texture changes of PEEK were found macroscopically. The clasp arm still fitted well without any deformation and a high subjective satisfaction was expressed by both the practitioner and the patient [39]. Further advantages include the low weight of PEEK prostheses, the tooth-similar color, a reportedly good fit and high retention [40, 41], and a protective effect on the periodontal ligament [42]. However, the indication of PEEK as a framework material remains controversial, as its stability in a free-end situation under masticatory forces is not conducive for a RDP’s stability [42].

While this study observed promising results for PEEK materials in regard to their potential use as RDP clasps and their high resistance against artificial aging in saliva, this in vitro study does entail several limitations. Only a small number of materials were tested in the present study. As the dental market moves quickly and new compositions are introduced each year, future studies will have to examine a wider range of materials. The use of artificial saliva furthermore only imitates one part of the manifold influences RDPs are exposed to during function, such as temperature changes or variations of the pH value. In the present study, only one clasp design, namely the popular Bonwill clasp, was examined. To allow the implementation of PEEK as a clasp material to a bigger extent, it is necessary to convey further examinations including a wide variety of clasp designs and geometries. As the environment has been reported to show varying effects on dislodging a clasp according to the type of clasp [43], and deformations differ due to the design of a clasp, future studies should focus on determining in how far PEEK materials could present a valid alternative to CoCrMo in specific situations, such as the esthetic anterior region presenting with little undercut, or periodontally damaged dentitions prone to the negative effects of excessively high retention forces [33]. The use of PEEK clasps could pave the way for a fully digital workflow in the treatment of patients with RDPs, from the digital impression to manufacturing using CAD/CAM technology [34]. Highly time- and resources-consuming laboratory processes in the fabrication of CoCrMo clasps that due to their manual background are furthermore prone to mistakes could hereby be replaced by machine processing of PEEK materials ensuring a high homogeneity of the material and promising great esthetic results. Moreover, due to the high surface resistance of PEEK material, its low reactivity, and a highly inert behavior in the oral cavity, these materials could have a good prognosis for allergy prone patients [19, 24]. Further clinical as well as laboratory studies are necessary to confirm the present findings.

## Conclusions

Within the limitations of this study, the following conclusions can be drawn:

1. Although the control group showed significantly higher results, the retention force values observed for PEEK materials indicate a potential clinical application.
2. The manufacturing process of PEEK did not influence the retention force.
3. While the control group was susceptible to artificial aging, PEEK materials presented constant results.
4. Ultimately, a repetitive insertion and removal of the clasps resulted in decreased retention force values.
